# Lockdown as a last resort option in case of COVID-19 epidemic rebound: a modelling study

**DOI:** 10.2807/1560-7917.ES.2021.26.22.2001536

**Published:** 2021-06-03

**Authors:** Cécile Tran Kiem, Pascal Crépey, Paolo Bosetti, Daniel Levy Bruhl, Yazdan Yazdanpanah, Henrik Salje, Pierre-Yves Boëlle, Simon Cauchemez

**Affiliations:** 1 Mathematical Modelling of Infectious Diseases Unit, Institut Pasteur, UMR2000, CNRS, Paris, France; 2 Collège Doctoral, Sorbonne Université, Paris, France; 3 Univ Rennes, EHESP, REPERES « Recherche en Pharmaco-Epidémiologie et Recours aux Soins » – EA 7449, Rennes, France; 4 Santé Publique France, French National Public Health Agency, Saint-Maurice, France; 5 Infections Antimicrobials Modelling Evolution (IAME), UMR1137, INSERM, University of Paris, Paris, France; 6 Department of Genetics, University of Cambridge, Cambridge, United Kingdom; 7 Institut Pierre Louis d’Epidémiologie et de Santé Publique, Sorbonne Université, INSERM, Paris, France

**Keywords:** epidemic modelling, COVID-19, air-borne infections, modelling, policy

## Abstract

**Background:**

Given its high economic and societal cost, policymakers might be reluctant to implement a large-scale lockdown in case of coronavirus disease (COVID-19) epidemic rebound. They may consider it as a last resort option if alternative control measures fail to reduce transmission.

**Aim:**

We developed a modelling framework to ascertain the use of lockdown to ensure intensive care unit (ICU) capacity does not exceed a peak target defined by policymakers.

**Methods:**

We used a deterministic compartmental model describing transmission of severe acute respiratory syndrome coronavirus 2 (SARS-CoV-2) and the trajectories of COVID-19 patients in healthcare settings, accounting for age-specific mixing patterns and an increasing probability of severe outcomes with age. The framework is illustrated in the context of metropolitan France.

**Results:**

The daily incidence of ICU admissions and the number of occupied ICU beds are the most robust indicators to decide when a lockdown should be triggered. When the doubling time of hospitalisations estimated before lockdown is between 8 and 20 days, lockdown should be enforced when ICU admissions reach 3.0–3.7 and 7.8–9.5 per million for peak targets of 62 and 154 ICU beds per million (4,000 and 10,000 beds for metropolitan France), respectively. When implemented earlier, the lockdown duration required to get back below a desired level is also shorter.

**Conclusions:**

We provide simple indicators and triggers to decide if and when a last-resort lockdown should be implemented to avoid saturation of ICU. These metrics can support the planning and real-time management of successive COVID-19 pandemic waves.

## Introduction

Given the high transmissibility and fatality rates associated with the severe acute respiratory syndrome coronavirus 2 (SARS-CoV-2) virus, a large number of countries implemented drastic lockdown strategies to avoid a complete saturation of their intensive care units (ICU). Like many other European countries, France implemented a lockdown of its population on 17 March 2020 [1], which led to a 77% drop in SARS-CoV-2 transmission rate and a reduction in daily ICU admissions from 700 in late March to 44 on 11 May [2]. The lockdown was then replaced by less restrictive physical distancing measures, the general use of face masks and the implementation of an approach based on the detection, testing and isolation of cases and their contacts. To cope with a rebound of the epidemic, a new lockdown was implemented in November 2020.

Thorough monitoring of the epidemic is essential to quickly detect possible epidemic rebounds and, if needed, implement corrective measures. When a local surge in cases has been identified, authorities in Germany, Portugal, the United Kingdom or Australia have not hesitated to quickly impose local lockdowns [3] affecting a few hundred thousand inhabitants so as to ensure spread is contained and spatial expansion avoided. However, given the major economic and societal costs associated with general lockdowns, the decision may be more difficult in scenarios where the number of cases grows slowly in a wide area. In such circumstances, authorities might prefer strengthening control measures without going as far as a general lockdown, for example with extended curfews [4], hoping this will be sufficient to contain spread at a lower cost for society. However, should these alternative control measures be unsuccessful, a new lockdown could be a last resort.

In this paper, we developed a modelling framework to help policymakers assess the use of lockdown as a last resort option, by determining when a lockdown should be adopted to avoid passing a predetermined ICU capacity target and by evaluating situations where a slowly growing epidemic might remain manageable for the healthcare system without the need for a lockdown. This was done for a broad range of rebound scenarios that are characterised by their doubling time (i.e. the time it takes for the daily number of cases to double) and exploring the many uncertainties that remain. We illustrated this framework in the context of metropolitan France.

## Methods

### Model for rebound scenarios

We adapted a mathematical model described in detail by Salje et al. [2]. In short, the model characterises the transmission of SARS-CoV-2 in the population of metropolitan France as well as its impact on healthcare capacity requirements in terms of ICU and general ward beds. It accounts for age-specific contact patterns and the increase of infection severity with age.

We characterised rebound scenarios with the effective reproduction number *R_eff_* at the beginning of a rebound, assuming that transmission rates remain stable until a lockdown is implemented. We considered scenarios in which this reproduction number ranges from 1.1 to 2.5.

For illustration, we assumed that the epidemic rebound started on 1 September 2020. For the time period between 11 May 2020 (when the first lockdown in France ended) and 1 September 2020, we assumed that the basic reproduction number was 0.9, consistent with what has been estimated in different European settings following the end of lockdowns [5]. As in Salje et al. [2], we worked with normalised contact matrices, so that changing the contact matrix only impacted the structure of contacts between age classes but not the effective reproduction number. For the time period between 11 May and 1 September 2020, we considered a contact matrix characterised by a reduction of 70% of contacts in schools, 50% of contacts in the workplace and 50% of contacts outside home and the workplace, compared with contact patterns before the beginning of the epidemic. From 1 September, we assumed a reduction of 30% of contacts in schools, 50% of contacts in the workplace and 50% of contacts outside home. The reduction in contacts at work is intended to capture both an increased frequency of teleworking [6] and the maintenance of preventive measures (e.g. physical distancing, use of face masks). In a sensitivity analysis, we also considered scenarios in which individuals older than 70 years further reduced their contacts by 20% or 40% instead of 0% assumed in our baseline scenario. Contact matrices were generated from the COMES-F survey [7] which describes age- and location-specific contact patterns in the French population, using the *socialmixr* package [8].

For each scenario, we defined the origin of time as the first day when the daily number of ICU admissions exceeds 10 at the national level (0.15 daily admissions/million inhabitants).

### Disease severity

We considered different disease severity scenarios that captured uncertainties about the probability of ICU admission given hospitalisation. In Salje et al. [2], we estimated the probability of ICU admission given hospitalisation by age group. We had found that the average probability decreased during the first wave of the COVID-19 pandemic, from 26.6% initially to 14.2% at the end of the wave, with an average probability of 19.0% [2]. This reduction summarises the effect of changing patient profiles, organisation and improvement in care. We therefore defined three scenarios for the probability of ICU admission given hospitalisation by age group (Supplementary Table S2) with an average of 14% in the medium scenario (estimate at the end of the first wave), of 19% in the high scenario (i.e. average estimate in first wave) and of 10% in the low scenario to account for the possibility of improved medical care or treatment. Unless stated otherwise, we consider the medium severity scenario in the following.

In Salje et al. [2], we had also estimated the probability of hospitalisation given infection for each age group (Supplementary Table S2), with an average probability equal to 2.9% (95% credible interval (CI): 1.7–4.8) during the first wave of COVID-19 in France. We here used the average central estimate of 2.9%, which is consistent with results from seroprevalence studies [9]. This probability is expected to vary by country with the age structure and prevalence of comorbidities [10]. To capture these situations, we also considered, in a sensitivity analysis, scenarios where the age-specific probability of hospitalisation given infection was equal to the lower bound (average = 1.7% during the first wave in France) and the upper bound (average = 4.8% during the first wave in France) obtained by Salje et al. [2] (Supplementary Table S2). In each of these scenarios, the average probability of hospitalisation given infection depended on age-specific attack rates and age-specific mixing patterns in the simulated scenario [2].

### Time spent in intensive care units and general wards

In our baseline analysis, we assumed that COVID-19 patients admitted to ICU spend 17.6 days on average in ICU, while COVID-19 patients not admitted to ICU spend 13.1 days in general wards, as observed in metropolitan France between 15 March and 7 May 2020 [2]. In a sensitivity analysis, we also considered scenarios where the time spent in ICU was 14.6 and 11.6 days.

### Simulations

For each rebound scenario, we simulated epidemic trajectories in the absence of lockdown or of other additional interventions to reduce transmission. We then simulated a lockdown starting between 1 September 2020 and 1 September 2021. We assumed that a lockdown would lead to the same decay rate of the epidemic as the one observed between 17 March and 11 May 2020. We computed the length of lockdown as the time necessary for the daily number of ICU admissions to return to levels similar to those measured on 11 May 2020 (0.7 per million inhabitants).

We determined the latest lockdown date that ensured that peak capacity for COVID-19 ICU beds would remain below a certain peak target to be defined by policymakers. Before the beginning of the COVID-19 epidemic, France had a capacity of 5,000 ICU beds (77 beds/million) that was temporarily increased to around 10,000 ICU beds (154 beds/million). However, increasing ICU bed capacity to 10,000 ICU beds comes with major negative impact for healthcare in France including the complete reshaping of healthcare services, the postponing of elective surgeries, the lack of healthcare for non-COVID-19 patients and the exhaustion of healthcare personnel that are already very affected by the pandemic. Allowing such high levels of viral circulation also implies a very large number of deaths. In our baseline scenario, we therefore report the results for a peak target of 4,000 ICU beds (62 ICU beds/million) so as to save 1,000 ICU beds for non-COVID19 patients and to allow the healthcare system to work as normally as possible. In a sensitivity analysis, we show results for a broad range of targets, from 20 to 200 ICU beds per million inhabitants, so that policymakers can select the target they are interested in. In a context of uncertainty, we identified the most robust criterion to determine when the lockdown should be decided to avoid saturation of ICU capacity. The criteria we considered were: daily number of ICU admissions, daily number of hospitalisations and number of general ward beds or of ICU beds based on the day the new lockdown is to be decided.

To account for the ongoing epidemic dynamics, we pragmatically computed at date t the average doubling time over the last 30 days as

$$\bar{D}(t)=\frac{log(2)\;\times \;30}{log[H(t)]\;-\;log[H(t\;-\;30)]}$$

where H(t) is the number of hospitalisations on day t. We report criteria values based on the doubling time calculated over the 30 days before lockdown implementation.

### Other sensitivity analysis

We also considered scenarios with shorter serial intervals for SARS-CoV-2 transmission (5 and 6 days instead of 7 in our baseline scenario). In this case, the reproduction number during the lockdown was re-estimated so that the model remained consistent with the decay rate observed during the first lockdown (17 March–11 May 2020) [2].

## Results

**Figure 1:**
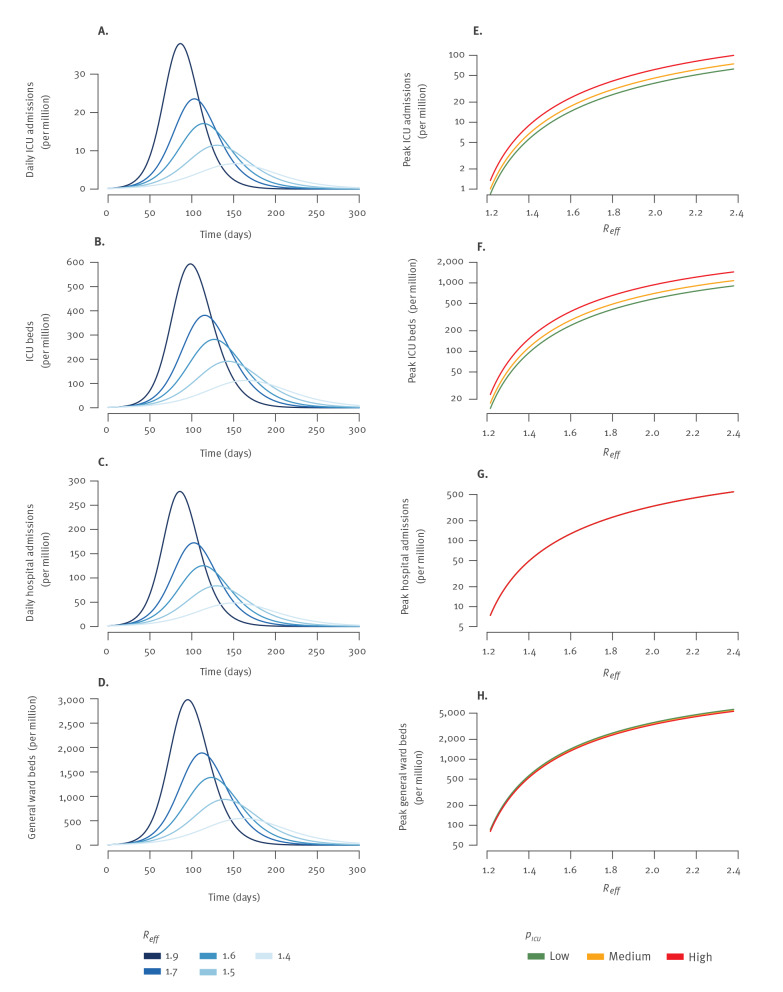
Trajectories in case of COVID-19 epidemic rebound that start with different values of the effective reproduction numbers and for different severity scenarios, France

COVID-19: coronavirus disease; ICU: intensive care unit; *p_ICU_*: probability of ICU admission given hospitalisation; *R_eff_*: effective reproduction number.

Figures 1A-D show the expected trajectory of healthcare demand for epidemic rebounds starting with different effective reproduction numbers assuming a constant transmission rate (i.e. no lockdown nor additional effective corrective measures) and in the scenario of medium severity. Time to peak in ICU beds was 165 days for an effective reproduction number initially at *R_eff_ *= 1.4 but 98 days for *R_eff_ *= 1.9 (Figure 1C, Supplementary Table S3). Healthcare demand at the peak was strongly impacted by values of the reproduction number and the severity scenario (Figure 1 E-H). For example, the number of ICU beds required at the peak was 593 per million inhabitants in the medium severity scenario (with a range of 497–795 for the different severity scenarios) for an initial reproduction number of *R_eff_ *= 1.9 and 113 per million inhabitants (range: 95–151) for a reproduction number of *R_eff_ *= 1.4 (Figure 1, Supplementary Table S3).

(A) Number of daily ICU admissions, (B) number of ICU beds occupied by COVID-19 patients, (C) number of daily hospital admissions and (D) number of general ward beds occupied by COVID-19 patients, for the medium severity scenario and different doubling times in the absence of lockdown. Peaks in (E) daily ICU admissions, (F) ICU beds, (G) daily hospital admissions and (H) general ward beds by doubling times of the epidemic. For panels E–H, the results are presented for different values of *p_ICU_*. In Panel (G), the lines for the three severity scenarios are superimposed.

Given the available ICU capacity, we estimated the highest initial effective reproduction number for which a lockdown could be avoided provided that the transmission rate does not increase over time. For example, in the scenario of medium severity, an ICU capacity of 62 beds per million (4,000 beds for metropolitan France) could cope with reproduction numbers < 1.2 without a lockdown. This result was moderately impacted by assumptions about severity. In the high severity scenario, this capacity could only cope with reproduction numbers < 1.17 (Supplementary Table S4).

**Figure 2:**
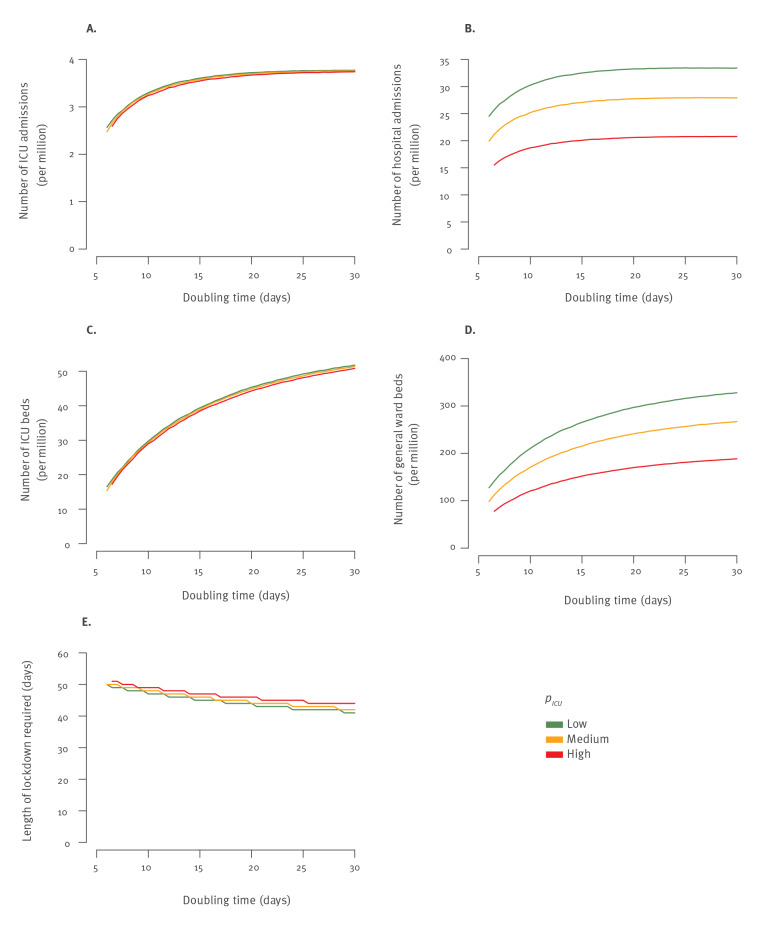
Timing and key indicators at the start of lockdown to remain below a peak capacity of 62 ICU beds per million inhabitants for different doubling times and severity scenarios, COVID-19 pandemic, France

COVID-19: coronavirus disease; ICU: intensive care unit; *p_ICU_*: probability of ICU admission given hospitalisation.

For each scenario, we determined the number of ICU admissions, hospitalisations and ICU and general ward beds occupied by COVID-19 patients on the day the lockdown should be implemented to remain below the peak ICU capacity target. We plotted these numbers as a function of the doubling time estimated in the 30 days before that day (Figure 2A-D). Considering the different criteria available to trigger a lockdown (Figures 2A-D, Supplementary Table S5), we found that the ones that were the least affected by uncertainty about severity were the daily number of ICU admissions and the number of ICU beds occupied (Supplementary Table S5). For example, for a doubling time of D = 10 days, the lockdown would need to be implemented when the daily number of ICU admissions reached 3.2–3.3 per million or when the number of ICU beds occupied by COVID-19 patients reached 29–30 for a peak target of ≤ 62 ICU beds per million. In contrast, other variables exhibited more variation, with hospital admissions ranging between 19 and 30 per million and the number of general ward beds occupied by COVID-19 patients between 121 and 211 per million.

(A) Number of COVID-19 ICU admissions, (B) number of COVID-19 hospital admissions, (C) number of ICU beds occupied with COVID-19 patients and (D) number of general ward beds occupied with COVID-19 patients on the day the lockdown is to be decided and (E) length of lockdown required to go back to the situation of 11 May 2020 in terms of ICU admissions. The doubling time is calculated over the 30 days before lockdown implementation. We present results for different values of *p_ICU_*.

**Figure 3:**
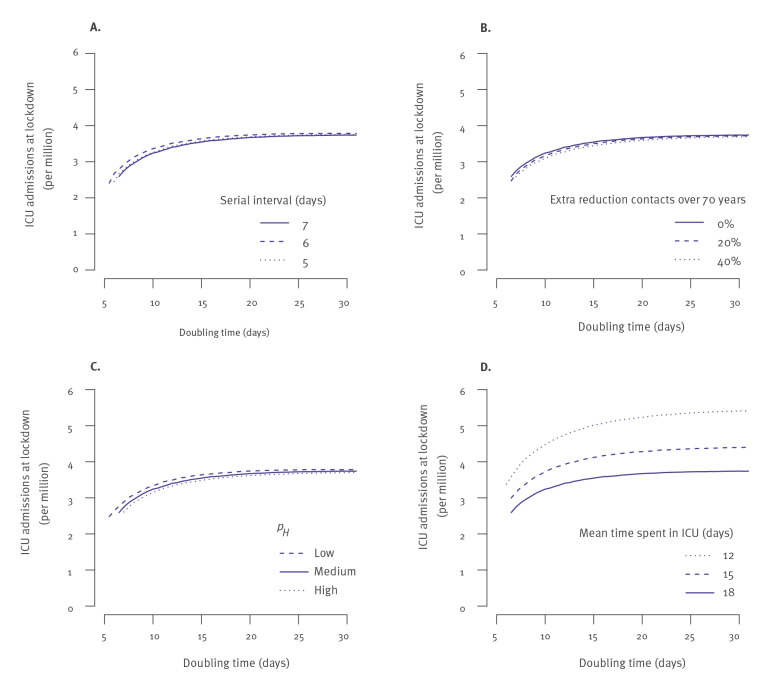
Sensitivity analysis: number of ICU admissions at the start of lockdown to avoid reaching a peak target of 62 ICU beds per million inhabitants for different doubling times of SARS-CoV-2 infections, France

COVID-19: coronavirus disease; ICU: intensive care unit; *pH*: probability of hospitalisation; SARS-CoV-2: severe acute respiratory syndrome coronavirus 2.

To ensure that needs in terms of ICU beds do not go above the peak target, we defined a simple decision rule where lockdown starts when the daily number of ICU admissions goes above the value identified in Figure 2A, considering the worst-case high-severity scenario. We found that this criterion was robust to a change in the serial interval (Figure 3A), to the contact patterns (Figure 3B) and to the probability of hospitalisation (Figure 3C) It was, however, sensitive to the length of stay in the ICU (Figure 3D, Table, Supplementary Tables S6-S7). For example, for a doubling time of 10 days, with a peak target of 62 ICU beds per million, the lockdown should be implemented when the daily number of ICU admissions reaches 4.5, 3.7 and 3.2 per million inhabitants for a length of stay in ICU of 11.6, 14.6 and 17.6 days, respectively. In the Table, we report criteria values for a broad range of doubling times and peak targets for ICU bed capacity so that decision makers can select the one they are interested in. Similar results for shorter lengths of stay in ICU (14.6 and 11.6 days) are reported in Supplementary Tables S6-S7.

Number of ICU admissions at lockdown (A) for different hypotheses about the serial interval of COVID-19, (B) for different contact matrices during the epidemic rebound period, (C) for different *p_H_* and (D) for different times spent in ICU. In panel B, we consider an additional reduction of contacts for individuals older than 70 years during the epidemic rebound period. The doubling time is computed over the last 30 days before lockdown implementation.

**Table:** Number of ICU admissions for COVID-19 per million on the day when a lockdown should be implemented to remain below a fixed peak target for ICU beds capacity, for a mean ICU stay of 17.6 days, France

Peak target for ICU beds capacity (per million)	Doubling time (days)						
	8	10	12	14	16	18	20
20	^a^	1	1.1	1.1	1.1	1.2	1.2
40	1.9	2.1	2.2	2.3	2.3	2.3	2.4
60	2.9	3.1	3.3	3.4	3.5	3.5	3.6
80	3.9	4.2	4.5	4.6	4.7	4.8	4.8
100	4.9	5.4	5.6	5.8	5.9	6	6
120	6	6.5	6.8	7	7.1	7.2	7.3
140	7.1	7.7	8	8.3	8.4	8.5	8.6
160	8.1	8.8	9.2	9.5	9.7	9.8	9.8
180	9.2	10	10.5	10.7	10.9	11.1	11.1
200	10.4	11.2	11.7	12	12.2	12.4	12.4

COVID-19: coronavirus disease; ICU: intensive care unit.

^a^ A lockdown should be implemented less than 30 days after the beginning of the timeline.

Calculated for different doubling times in the 30 days before lockdown implementation, assuming a mean length of stay in ICU of 17.6 days.

**Figure 4:**
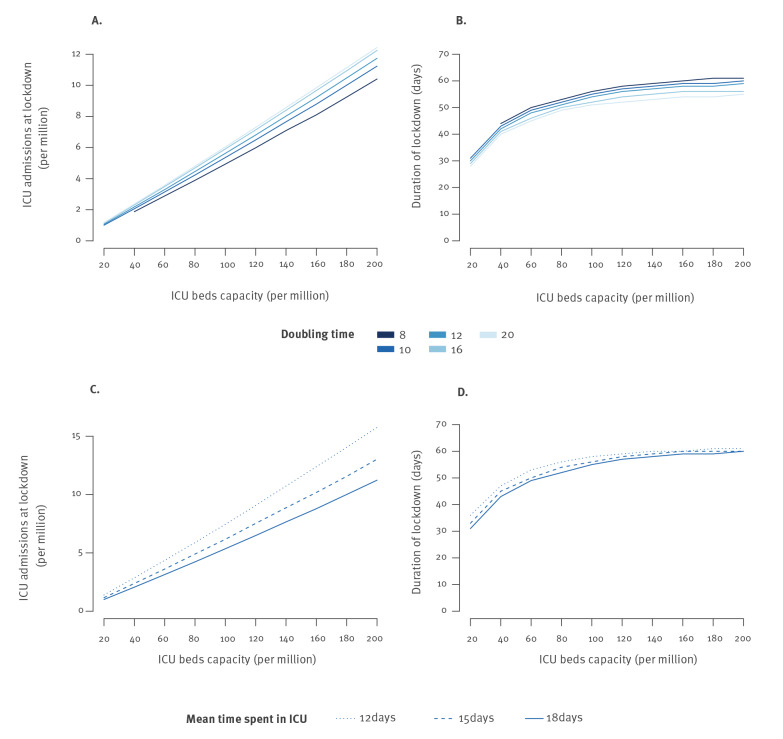
Number of ICU admissions for COVID-19 at the start of lockdown and duration of the lockdown as a function of the peak target for ICU bed capacity, France

COVID-19: coronavirus disease; ICU: intensive care unit; *p_ICU_*: probability of ICU admission given hospitalisation.

Figure 4 shows how the criterion changed as a function of the peak target for ICU bed capacity. For doubling times between 8 and 20 days, lockdown should be enforced when ICU admissions reach 3.0–3.7 and 7.8–9.5 per million inhabitants for peak targets of 62 and 154 beds per million, respectively (Figure 4 A-B). The duration of the lockdown strongly depended on the time when it was enforced. For a doubling time of 10 days, a lockdown enforced at 2.1 ICU admissions per million that satisfied a peak target of 40 ICU beds per million would last 43 days. By contrast, a lockdown enforced at 10.0 ICU admissions per million for a peak target of 180 ICU beds per million would last 59 days (Figure 4A-B). This trend remained unchanged when considering different lengths of stay in ICU (Figure 4C-D).

(A) ICU admissions at lockdown and (B) duration of lockdown for different doubling times of the epidemic. (C) ICU admissions at lockdown and (D) duration of lockdown for different lengths of stay in ICU. We assume that the lockdown stops when the number of ICU admissions drops below 0.68 per million inhabitants (number of ICU admissions observed on 11 May 2020 in metropolitan France, when the first lockdown stopped). The doubling time is computed over the last 30 days before lockdown implementation.

**Figure 5:**
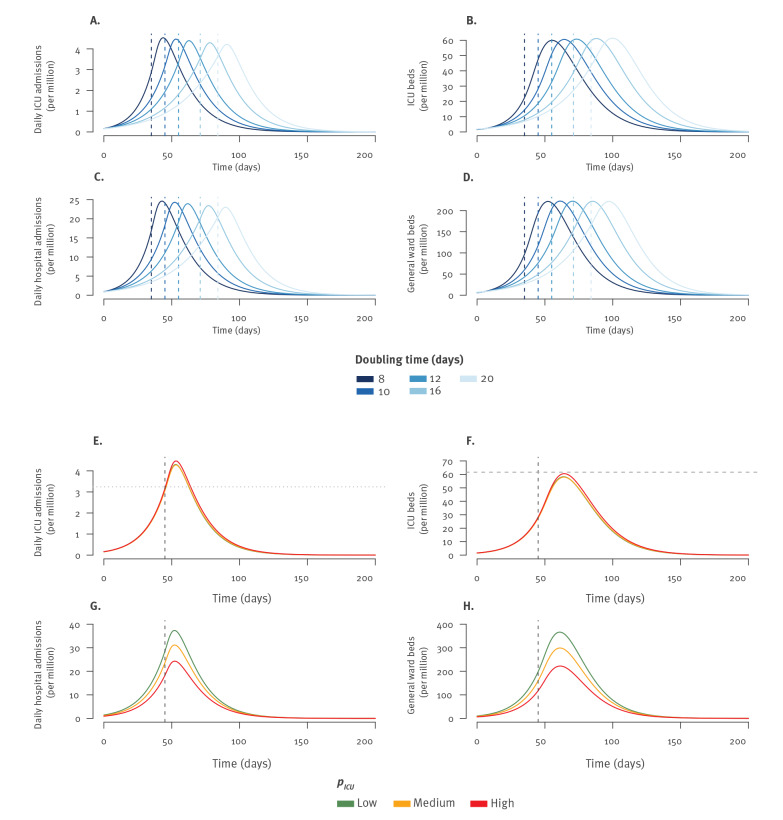
Trajectories for different lockdown scenarios to remain below a peak target capacity of 62 ICU beds per million inhabitants, COVID-19 pandemic, France

COVID-19: coronavirus disease; ICU: intensive care unit; *p_ICU_*: probability of ICU admission given hospitalisation.

Figure 5 shows the epidemic dynamics we obtained if a lockdown is decided based on our decision rule, for a peak target of 62 ICU beds per million. Applying the same rule irrespective of the severity scenario, we obtained similar peaks for the number of ICU beds (Figure 5E-F).

(A) Number of daily ICU admissions, (B) number of ICU beds occupied by COVID-19 patients, (C) number of daily hospital admissions and (D) number of general ward beds occupied by COVID-19 patients for different doubling times and in the medium severity scenario. (E) Daily ICU admissions, (F) number of ICU beds occupied by COVID-19 patients, (G) daily hospital admissions and (H) number of general wards beds occupied by COVID-19 patients for a doubling time of 10 days, for different values of *p_ICU_*.

The vertical dotted lines indicate the lockdown day decided with the criterion relying on ICU admissions to ensure that peak capacity does not go beyond 62 ICU beds per million inhabitants. The origin for the x-axis corresponds to the first day when the number of daily ICU admission exceeds 0.15 per million inhabitants. The horizontal grey dotted line in panel E corresponds to the number of daily ICU admissions on lockdown day. The horizontal grey dotted line in panel F corresponds to the ICU beds threshold occupied by COVID-19 patients of 62 beds per million inhabitants. The doubling time is computed over the last 30 days before lockdown implementation. Lockdowns are assumed to be implemented until the end of the simulation.

While the criterion to impose a lockdown was robust to assumptions about mixing patterns (Figure 3B), healthcare demand at the peak of the pandemic in the absence of lockdown was sensitive to these assumptions (Supplementary Figure S1). For example, if individuals older than 70 years further reduced their contacts by 40%, the mean probability of hospitalisation would decrease from 2.8% (range: 1.6–4.6%) to 2.3% (range: 1.3–3.8%) (Supplementary Table S8). For an initial effective reproduction number of 1.9, this would decrease ICU bed requirements at the peak from 593 to 485 per million (Supplementary Figure S1, Supplementary Tables S3, S9, S10) in the medium severity scenario. This would also increase our ability to cope with quickly growing epidemics (Supplementary Figure S2, Supplementary Tables S3, S11, S12).

## Discussion

Since the start of the pandemic, countries have heavily invested in the development of control strategies that aim to contain the spread of SARS-CoV-2 while limiting as much as possible their impact on economic and social activities. However, given the high transmissibility of SARS-CoV-2, the low proportion of individuals who developed antibodies [11-13] during the first wave means that lockdowns still had a role to play in the control of the pandemic in autumn 2020. Lockdowns have been used in two different situations [3]. Firstly, there are targeted lockdowns aiming to control localised outbreaks. Such lockdowns are generally triggered based on evidence of transmission clusters or if the density or number of persons testing positive crosses a certain threshold; the key for their success is speed of implementation and they are adopted for a limited duration. Secondly, there are general lockdowns that cover a wider area (potentially the whole country) and are decided to avoid the collapse of the health system. Such general lockdowns come at a very high societal and economic cost. As a consequence, policymakers may decide to adopt them if other control measures are thought to be insufficient. In this paper, we focused on the study of general lockdowns as a last resort option to avoid saturation of ICU capacity.

Our analysis shows that qualitatively, epidemic rebounds would fall into two categories. Those with low reproduction numbers could be accommodated under normal ICU bed capacity (Supplementary Table S4). However, as reproduction numbers increase, more explosive epidemic spread ensues and further interventions would be required to avoid saturation of hospital capacities. Even if epidemic growth is initially slow, it may accelerate over time and become less manageable for example because of a saturation of resources for testing and contact tracing, increased risk of superspreading events, changing climate conditions [11] or spatial expansion. An increase in the frequency of more transmissible variants [14,15] could also result in an acceleration of the epidemic. For all these reasons, it is important to respond robustly and promptly to any rebound, even those that start slowly. Furthermore, if there is clear indication that corrective measures are not sufficiently attenuating the epidemic and that a lockdown will therefore be necessary, the lockdown should be implemented promptly without waiting until the last minute. This is because our results show that early lockdown implementation is more effective as it leads to smaller peaks and can be lifted more quickly.

As expected, we found that, in the absence of a lockdown, healthcare demand at the peak critically depends on both the reproduction number of the epidemic and assumptions about the severity of infection. We therefore considered different scenarios to account for current uncertainties in these quantities as well as changes that could occur in future pandemic waves. Firstly, we used the whole range of probabilities estimated during the first pandemic wave to parametrise scenarios. Secondly, we studied scenarios with lower probability of admission to ICU and length of stay in ICU to account for potential improved patient care [12,13]. We also considered the possibility that older individuals would self-isolate more than younger people, leading to reduced healthcare demand at the peak (Supplementary Figure S1, Supplementary Tables S9-S10). Despite uncertainties surrounding healthcare demand at the peak (Supplementary Figure S1), we found that the observed incidence of ICU admissions was a robust indicator to determine the timing of a last-resort lockdown. Indeed, anticipating when a certain ICU bed capacity will be reached only requires predicting trends in ICU admissions from daily incidence and doubling times and trends in ICU discharge from known length of stay. By relying on observed incidence of ICU admissions, we found that we could control for model uncertainty about this sensitive variable.

As an example, a second nationwide lockdown of the French population was implemented on 30 October 2020 when the number of ICU admissions reached 5.9 per million (Supplementary Figure S3). At that date, hospital admissions had doubled on average every 13 (range: 12–15) days in the preceding month. According to our framework, for such a doubling time, the lockdown would be expected to limit the number of required ICU beds to less than 75 (range: 73–76) per million for a mean length of stay of 11.6 days in ICU and to 90 (range: 88–92) per million for a mean length of stay of 14.6 days. These predictions are in excellent agreement with the 76 ICU beds per million that were eventually occupied by COVID-19 patients at the peak in the beginning of November 2020 (Supplementary Figure S3). Our model relied on a number of assumptions. Determining how contacts are structured when control measures are implemented remains difficult. In most countries, physical distancing measures are still ongoing, although a gradual lifting of measures is taking place in a number of settings. As we worked with normalised contact matrices [2], modifying the contact matrix mostly impacted the structure of contacts between age classes but not the doubling time which is the key parameter of interest here. Estimates of the mean serial interval ranged between 4 and 8 days [16-18], where earlier detection and isolation of infectious individuals may explain the shorter durations [19]. This uncertainty affects estimates of the reproduction number (Supplementary Table S1) [20]. Hence, we determined our criteria for lockdown implementation according to the epidemic doubling time measured over the 30 days before lockdown decision which can be measured without further hypotheses. This pragmatic approach can also help capture temporal variations in the transmission rate that are expected in such an epidemic.

We used the situation in metropolitan France as a template for analysis. Our results may serve as a guide for other populations that have similar demographics, such as neighbouring European countries or French overseas territories such as Guadeloupe or Martinique. However, since age is a key determinant of the severity of infection, our model would have to be rerun for populations with different age pyramids. In particular, for a given doubling time, populations with younger demographics such as French Guiana will have substantially lower ICU needs at the peak [4]. The probability of ICU admission given hospitalisation and the probability of hospital admission upon infection may vary by country. To support assessment in these locations, we considered a broad range of severity parameters that could capture part of these heterogeneities. Our model suggests that ca 5% of the French population has been infected by SARS-CoV-2 during the first pandemic wave [2], which has since been confirmed by seroprevalence studies [13,21]. Our framework should still be able to provide meaningful results in regions that have higher levels of immunity. This is because more immunity would probably translate into longer doubling times, which our approach controls for. Our approach was designed at a time where no vaccine was available. In settings that have reached high vaccination coverages, it is expected that the control of the pandemic can be successful without the implementation of lockdowns. Our results could be used to define strategies at a regional scale although stochastic effects may play a larger role in smaller areas.

## Conclusion

We presented a framework to evaluate the use of lockdown as a last resort option to avoid saturation of ICU in the context of a COVID-19 epidemic rebound in settings with low levels of vaccination coverage. Our results can be used to inform planning for successive COVID-19 pandemic waves.

## Supplementary Data

735000 Supplement
