# Supplementary material for: Lockdown as a last resort option in case of COVID-19 epidemic rebound: a modelling study
Source: Euro Surveill. 2021 Jun 3;26(22):2001536. doi: 10.2807/1560-7917.ES.2021.26.22.2001536 (PMC8176673; doi:10.2807/1560-7917.ES.2021.26.22.2001536)
Supplement: Supplement [file 20-01536_CAUCHEMEZ_Supplement.pdf]

Supplementary information for:

Lockdown as a last resort option in case of COVID-19 epidemic rebound: a modelling study;

Cécile Tran Kiem[1,2], Pascal Crépey[3], Paolo Bosetti[1], Daniel Levy Bruhl[4], Yazdan Yazdanpanah[5], Henrik Salje[1,6], Pierre-Yves Boëlle[7], Simon Cauchemez[1]

1. Mathematical Modelling of Infectious Diseases Unit, Institut Pasteur, UMR2000, CNRS, Paris, France
2. Collège Doctoral, Sorbonne Université, Paris, France
3. Univ Rennes, EHESP, REPERES « Recherche en Pharmaco-Epidémiologie et Recours aux Soins » – EA 7449, Rennes, France
4. Santé Publique France, French National Public Health Agency, Saint-Maurice, France
5. Infections Antimicrobials Modelling Evolution (IAME), UMR1137, INSERM, University of Paris, Paris, France
6. Department of Genetics, University of Cambridge, Cambridge, United Kingdom
7. Institut Pierre Louis d'Epidémiologie et de Santé Publique, Sorbonne Université, INSERM, Paris, France

This supplementary material is hosted by Eurosurveillance as supporting information alongside the article **Lockdown as a last resort option in case of COVID-19 epidemic rebound: A modelling study**, on behalf of the authors, who remain responsible for the accuracy and appropriateness of the content. The same standards for ethics, copyright, attributions and permissions as for the article apply. Supplements are not edited by Eurosurveillance and the journal is not responsible for the maintenance of any links or email addresses provided therein.

Content :

Table S1-S12

Figure S1-S3

Table S1: Correspondence table between doubling times and effective reproduction numbers under our assumptions for the natural history of COVID-19. In our baseline model, we assume that i) infected individuals become infectious after on average 4 days, ii) those who develop symptoms will develop them on average 1 day after the beginning of infectiousness, iii) individuals are infectious on average for 4 days.

Doubling time (days)	Effective reproduction number		
	Serial Interval 7 days (baseline model)	Serial Interval 6 days	Serial Interval 5 days
5	2.27	2.07	1.88
6	2.02	1.86	1.72
7	1.85	1.72	1.61
8	1.73	1.62	1.52
10	1.57	1.49	1.41
12	1.46	1.40	1.34
14	1.39	1.34	1.29
16	1.34	1.29	1.25
18	1.30	1.26	1.22
25	1.21	1.18	1.16
30	1.17	1.15	1.13

Table S2: Age-dependent probability of ICU admission given hospitalisation used in the different severity scenarios and age-dependent probability of hospitalisation given infection used in the sensitivity analyses. Age-dependent severity parameters are derived from Salje et al.[1]

Age	p_{ICU}			p_H		
	Low	Medium	High	Low	Medium	High
0-19	14.0%	16.7%	22.2%	0.08%	0.1%	0.2%
20-29	7.3%	8.7%	11.6%	0.3%	0.5%	0.8%
30-39	10.0%	11.9%	15.9%	0.6%	1.1%	1.7%
40-49	14.0%	16.7%	22.2%	0.8%	1.4%	2.3%
50-59	17.4%	20.8%	27.6%	1.6%	2.9%	4.7%
60-69	19.4%	23.2%	30.8%	3.3%	5.8%	9.5%
70-79	15.7%	18.8%	24.9%	5.2%	9.3%	15.1%
80+	3.5%	4.2%	5.6%	14.7%	26.2%	42.7%

Table S3: Occupation of ICU beds without lockdown. Number of ICU beds at the peak and timing of the peak in ICU beds for different initial effective reproduction number of the epidemic and different severity scenarios characterized the probability of ICU admission given hospitalisation p_{ICU}. The timing of the peak in ICU beds is measured starting from the day when the number of daily ICU admissions exceeds 0.15 per million.

		ICU beds at the peak (per million inhabitants)			Timing of the peak in ICU beds (days)		
	p_{ICU}	Low	Medium	High	Low	Medium	High
Initial effective reproduction number	1.9	497	593	795	96	98	102
	1.8	410	489	655	103	105	110
	1.7	319	381	511	112	115	120
	1.6	236	282	378	124	127	133
	1.5	161	192	257	139	143	150
	1.4	95	113	151	160	165	174
	1.3	43	52	69	193	200	213

Table S4: Lowest initial effective reproduction number for which the number of ICU beds at the peak will not go above target. This is calculated in the absence of a lockdown. Results are reported for different severity scenarios regarding the probability of ICU admission given hospitalisation.

Peak target for ICU bed capacity per million inhabitants (number for metropolitan France)	Probability of ICU admission given hospitalisation p_{ICU}		
	Low	Medium	High
46 (3,000)	1.18	1.17	1.15
62 (4,000)	1.21	1.2	1.17
77 (5,000)	1.24	1.22	1.19
108 (7,000)	1.29	1.26	1.22
123 (8,000)	1.31	1.28	1.24
154 (10,000)	1.35	1.32	1.27
216 (14,000)	1.42	1.38	1.32
247 (16,000)	1.46	1.41	1.35

Table S5: Key indicators measured on the last day when a lockdown should be implemented to ensure that the number of ICU beds does not go beyond 62 per million inhabitants. Key indicators include the time to lockdown, the daily number of ICU admissions and of hospital admissions, the number of ICU beds, the number of general ward beds and the length of the lockdown. Time to lockdown is measured from the day when the daily number of ICU admissions exceeds 0.15 per million. We assume that the lockdown stops when the number of ICU admission goes back to the situation on May 11th (0.7 per million). Results are reported for different severity scenarios regarding the probability of ICU admission given hospitalisation p_{ICU}. The doubling time is computed over the last 30 days before lockdown implementation.

	p_{ICU}	Doubling time (days)					
		8	10	12	14	16	20
ICU admissions per million inhabitants at lockdown	Low	3	3.3	3.5	3.6	3.6	3.7
	Medium	3	3.3	3.4	3.5	3.6	3.7
	High	3	3.2	3.4	3.5	3.6	3.7
Hospital admissions per million inhabitants at lockdown	Low	28.2	30.3	31.5	32.2	32.7	33.3
	Medium	23.5	25.2	26.2	26.9	27.3	27.7
	High	17.3	18.7	19.5	19.9	20.3	20.6
ICU beds per million inhabitants at lockdown	Low	23.9	29.7	34.2	37.7	40.7	45.4
	Medium	23.5	29.3	33.9	37.5	40.4	44.9
	High	23	29	33.4	36.9	39.9	44.3
General ward beds per million inhabitants at lockdown	Low	175.3	210.6	236.5	256.1	272.5	297
	Medium	141.7	170.8	192.4	209	222.2	241.2
	High	99.4	120.7	135.7	147.2	156.4	170
Length of lockdown required (days)	Low	48	47	46	46	45	44
	Medium	49	48	47	46	46	44
	High	50	49	48	47	47	46

Table S6: Number of ICU admissions per million on the day when a lockdown should be implemented to satisfy a certain peak target for ICU beds capacity, for a mean sojourn time in ICU of 14.6 days. This is calculated for different doubling times over the last 30 days prior lockdown implementation. (-) indicates when a lockdown should be implemented less than 30 days after the beginning of the timeline.

Peak target for ICU beds capacity (per million)	Doubling time (days)						
	8	10	12	14	16	18	20
20	-	1.2	1.2	1.3	1.3	1.3	1.4
40	2.2	2.4	2.5	2.6	2.7	2.7	2.8
60	3.3	3.6	3.8	4	4.1	4.1	4.2
80	4.5	4.9	5.1	5.3	5.5	5.5	5.6
100	5.7	6.2	6.5	6.7	6.9	7	7.1
120	6.9	7.5	7.9	8.2	8.3	8.4	8.5
140	8.1	8.9	9.3	9.6	9.8	9.9	10
160	9.4	10.2	10.7	11.1	11.3	11.4	11.5
180	10.6	11.6	12.1	12.5	12.8	12.9	13
200	12	13	13.6	14	14.3	14.5	14.5

Table S7: Number of ICU admissions per million on the day when a lockdown should be implemented to satisfy a certain peak target for ICU beds capacity, for a mean sojourn time in ICU of 11.6 days. This is calculated for different doubling times over the last 30 days prior lockdown implementation. (-) indicates when a lockdown should be implemented less than 30 days after the beginning of the timeline.

Peak target for ICU beds capacity (per million)	Doubling time (days)							
	6	8	10	12	14	16	18	20
20	-	1.2	1.4	1.5	1.6	1.6	1.6	1.7
40	-	2.6	2.9	3	3.2	3.2	3.3	3.4
60	3.3	4	4.4	4.6	4.8	4.9	5	5.1
80	4.5	5.4	5.9	6.2	6.5	6.7	6.8	6.9
100	5.7	6.8	7.4	7.9	8.2	8.4	8.5	8.6
120	7	8.3	9.1	9.6	9.9	10.2	10.3	10.5
140	8.4	9.8	10.7	11.3	11.7	12	12.2	12.3
160	9.6	11.4	12.4	13.1	13.5	13.8	14	14.2
180	11.1	12.9	14.1	14.9	15.3	15.6	15.9	16
200	12.4	14.6	15.8	16.7	17.2	17.5	17.7	17.9

Table S8: Mean probability of hospitalisation upon infection for different mixing patterns

Additional reduction of contacts in individuals aged ≥70y	Mean probability of hospitalisation upon infection for Medium (Low-High) severity	Mean probability of ICU admission given hospitalisation for Medium (Low-High) severity
0%	2.84% (1.60% - 4.63%)	13.79% (11.56% - 18.32%)
20%	2.58% (1.45%- 4.21%)	14.31% (11.99% - 19.00%)
40%	2.31% (1.30% - 3.76%)	14.99% (12.56% - 19.90%)

Table S9 Characteristics of the peak in ICU beds without the implementation of a lockdown assuming individuals aged >70 additionally reduce their contacts by 20%. Number of ICU beds at the peak and timing of the peak in ICU beds for different initial doubling times of the epidemic and different probabilities of ICU admission given hospitalisation p_{ICU}. The timing of the peak in ICU beds is measured starting from the day when the number of daily ICU admissions exceeds 0.15 per million.

		ICU beds at the peak (per million inhabitants)			Timing of the peak in ICU beds (days)		
	p_{ICU}	Low	Medium	High	Low	Medium	High
Initial effective reproduction number	1.9	451	538	721	95	97	101
	1.8	370	442	592	101	104	108
	1.7	288	344	460	111	114	119
	1.6	212	253	339	122	125	131
	1.5	144	172	230	136	141	147
	1.4	85	101	135	157	162	171
	1.3	39	46	62	187	195	207

Table S10: Characteristics of the peak in ICU beds without the implementation of a lockdown assuming individuals aged >70 additionally reduce their contacts by 40%. Number of ICU beds at the peak and timing of the peak in ICU beds for different initial doubling times of the epidemic and different probabilities of ICU admission given hospitalisation p_{ICU}. The timing of the peak in ICU beds is measured starting from the day when the number of daily ICU admissions exceeds 0.15 per million.

		ICU beds at the peak (per million inhabitants)			Timing of the peak in ICU beds (days)		
	p_{ICU}	Low	Medium	High	Low	Medium	High
Initial effective reproduction number	1.9	406	485	649	94	96	100
	1.8	333	398	533	100	103	107
	1.7	259	309	414	109	112	117
	1.6	190	227	305	120	123	129
	1.5	129	154	206	134	138	145
	1.4	76	91	121	154	159	168
	1.3	35	41	56	183	190	203

Table S11: Highest initial effective reproduction number for which the number of ICU beds at the peak will not go above target assuming individuals aged >70 additionally reduce their contacts by 20%. This is calculated in the absence of a lockdown. Results are reported for different probabilities of ICU admission given hospitalisation p_{ICU}.

Peak target for ICU bed capacity per million inhabitants (number for metropolitan France)	Probability of ICU admission given hospitalisation p_{ICU}		
	Low	Medium	High
46 (3,000)	1.2	1.18	1.15
62 (4,000)	1.23	1.21	1.18
77 (5,000)	1.26	1.23	1.2
108 (7,000)	1.3	1.28	1.24
123 (8,000)	1.33	1.3	1.26
154 (10,000)	1.37	1.34	1.29
216 (14,000)	1.45	1.4	1.34
247 (16,000)	1.49	1.44	1.37

Table S12: Highest initial effective reproduction number for which the number of ICU beds at the peak will not go above target assuming individuals aged >70 additionally reduce their contacts by 40%. This is calculated in the absence of a lockdown. Results are reported for different probabilities of ICU admission given hospitalisation p_{ICU}.

Peak target for ICU bed capacity per million inhabitants (number for metropolitan France)	Probability of ICU admission given hospitalisation p_{ICU}		
	Low	Medium	High
46 (3,000)	1.21	1.19	1.16
62 (4,000)	1.24	1.22	1.19
77 (5,000)	1.27	1.25	1.21
108 (7,000)	1.32	1.29	1.25
123 (8,000)	1.35	1.32	1.27
154 (10,000)	1.4	1.36	1.3
216 (14,000)	1.48	1.43	1.37
247 (16,000)	1.52	1.47	1.4

Figure S1: Sensitivity analyses of peak characteristics in the absence of lockdown considering alternative mixing patterns where individuals aged >70y further reduce their contacts by 20% and 40% (0%: baseline). (A) Daily ICU admissions, **(B)** ICU beds, **(C)** daily hospital admissions and **(D)** general ward beds peak characteristics for different doubling times. Results are reported for different probabilities of ICU admission given hospitalisation p_{ICU}.

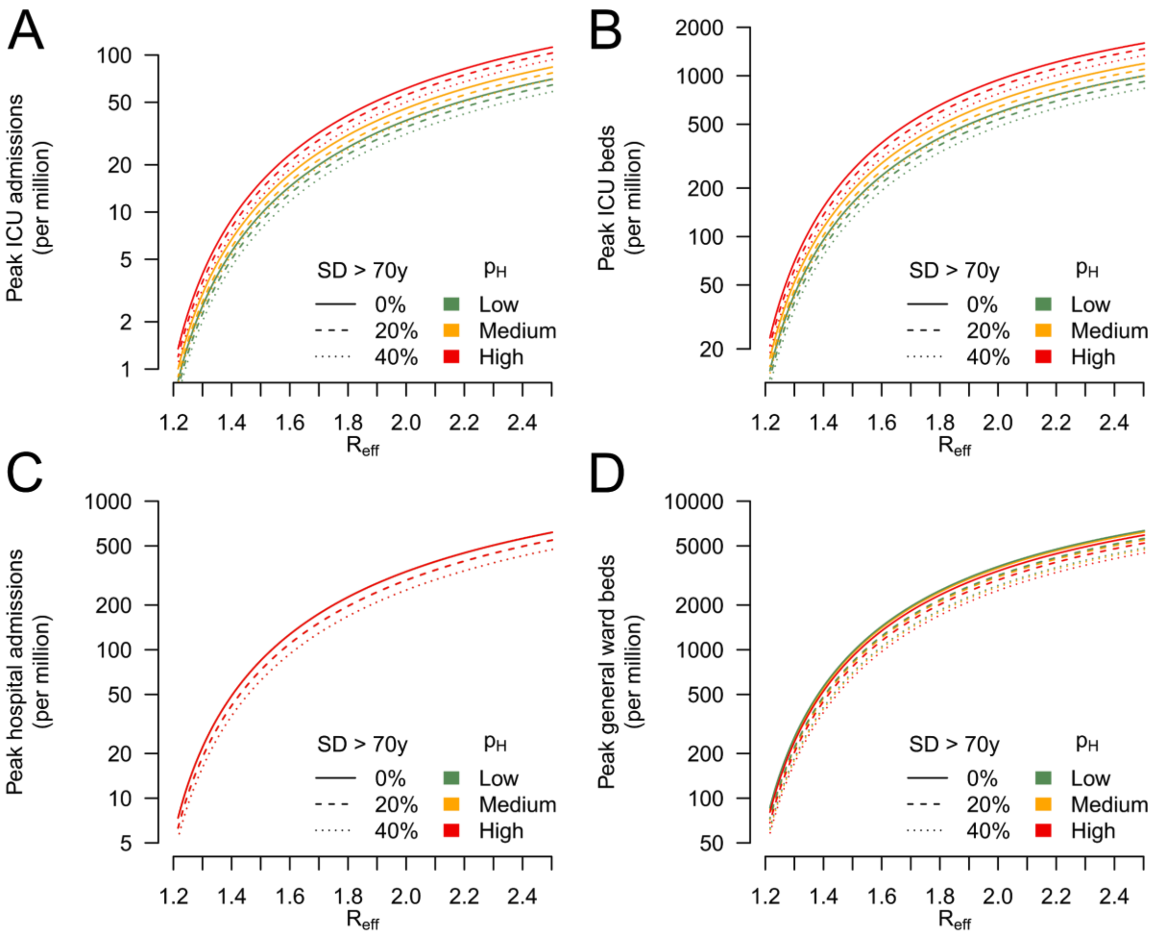

Figure S2: Highest sustainable effective reproduction number as a function of ICU peak target capacity considering alternative mixing patterns where individuals aged >70y further reduce their contacts by 20% and 40% (0%: baseline). Results are reported for different probabilities of ICU admission given hospitalisation p_{ICU}.

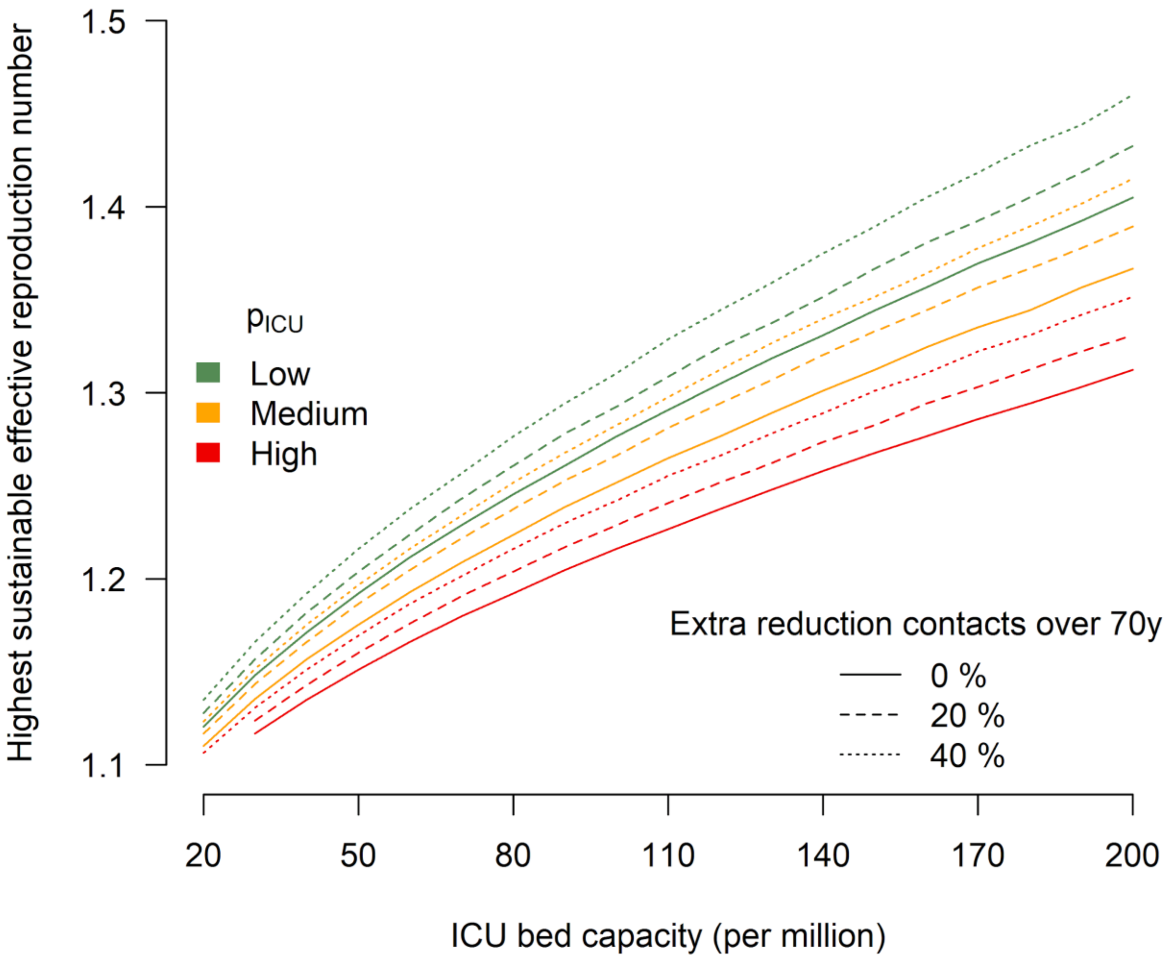

Figure S3: Hospital admissions, ICU admissions and number of beds occupied by COVID-19 patients in metropolitan France. (A) Hospital admissions (per million inhabitants) reported in the SI-VIC database. The doubling time is computed from the growth rate of hospital admissions (red line). **(B)** ICU admissions (per million) reported in the SI-VIC database. The grey line corresponds to the 7-day moving average. The vertical dotted line corresponds to the day the second nationwide lockdown was decided. **(C)** Number of ICU beds reported in the SI-VIC database. The SI-VIC database in a national surveillance system providing real time estimates on the COVID-19 patients hospitalized in public and private French hospitals. Numbers are corrected for reporting delays as described elsewhere.[1]

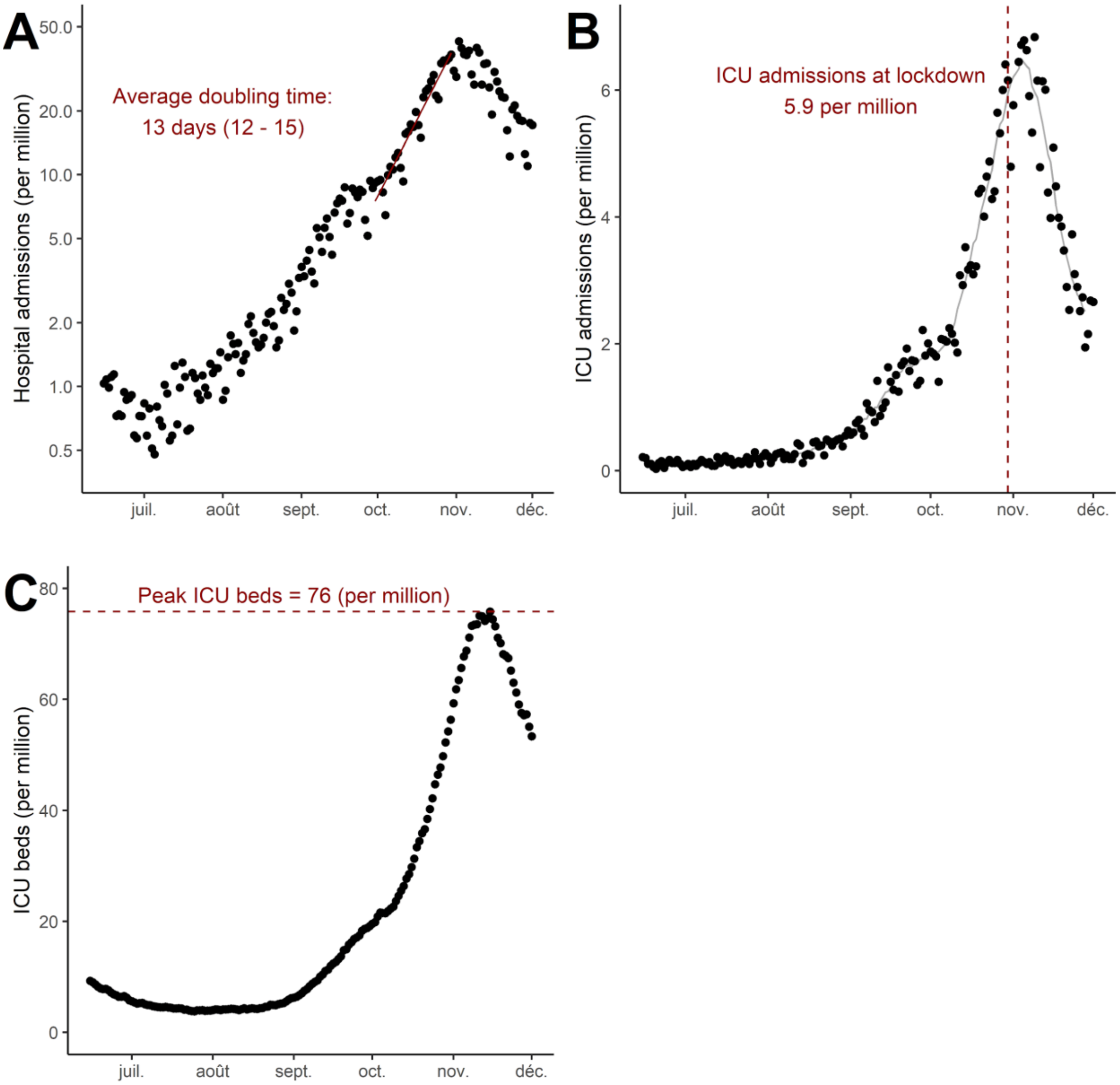

References

1. Salje, H. *et al.* Estimating the burden of SARS-CoV-2 in France. *Science* **369**, 208–211 (2020).
